# Study and Investigation on 5G Technology: A Systematic Review

**DOI:** 10.3390/s22010026

**Published:** 2021-12-22

**Authors:** Ramraj Dangi, Praveen Lalwani, Gaurav Choudhary, Ilsun You, Giovanni Pau

**Affiliations:** 1 School of Computing Science and Engineering, VIT University Bhopal, Bhopal 466114, India; ramraj.dangi2019@vitbhopal.ac.in (R.D.); praveen.lalwani@vitbhopal.ac.in (P.L.); 2 Department of Applied Mathematics and Computer Science, Technical University of Denmark, 2800 Lyngby, Denmark; gauravchoudhary7777@gmail.com; 3 Department of Information Security Engineering, Soonchunhyang University, Asan-si 31538, Korea; 4 Faculty of Engineering and Architecture, Kore University of Enna, 94100 Enna, Italy; giovanni.pau@unikore.it

**Keywords:** 5G, millimeter wave (mmW), massive multiple input and multiple output (MIMO), small cell, mobile edge computing (MEC), beamforming, machine learning

## Abstract

In wireless communication, Fifth Generation (5G) Technology is a recent generation of mobile networks. In this paper, evaluations in the field of mobile communication technology are presented. In each evolution, multiple challenges were faced that were captured with the help of next-generation mobile networks. Among all the previously existing mobile networks, 5G provides a high-speed internet facility, anytime, anywhere, for everyone. 5G is slightly different due to its novel features such as interconnecting people, controlling devices, objects, and machines. 5G mobile system will bring diverse levels of performance and capability, which will serve as new user experiences and connect new enterprises. Therefore, it is essential to know where the enterprise can utilize the benefits of 5G. In this research article, it was observed that extensive research and analysis unfolds different aspects, namely, millimeter wave (mmWave), massive multiple-input and multiple-output (Massive-MIMO), small cell, mobile edge computing (MEC), beamforming, different antenna technology, etc. This article’s main aim is to highlight some of the most recent enhancements made towards the 5G mobile system and discuss its future research objectives.

## 1. Introduction

Most recently, in three decades, rapid growth was marked in the field of wireless communication concerning the transition of 1G to 4G [1,2]. The main motto behind this research was the requirements of high bandwidth and very low latency. 5G provides a high data rate, improved quality of service (QoS), low-latency, high coverage, high reliability, and economically affordable services. 5G delivers services categorized into three categories: (1) Extreme mobile broadband (eMBB). It is a nonstandalone architecture that offers high-speed internet connectivity, greater bandwidth, moderate latency, UltraHD streaming videos, virtual reality and augmented reality (AR/VR) media, and many more. (2) Massive machine type communication (eMTC), 3GPP releases it in its 13th specification. It provides long-range and broadband machine-type communication at a very cost-effective price with less power consumption. eMTC brings a high data rate service, low power, extended coverage via less device complexity through mobile carriers for IoT applications. (3) ultra-reliable low latency communication (URLLC) offers low-latency and ultra-high reliability, rich quality of service (QoS), which is not possible with traditional mobile network architecture. URLLC is designed for on-demand real-time interaction such as remote surgery, vehicle to vehicle (V2V) communication, industry 4.0, smart grids, intelligent transport system, etc. [3].

### 1.1. Evolution from 1G to 5G

First generation (1G): 1G cell phone was launched between the 1970s and 80s, based on analog technology, which works just like a landline phone. It suffers in various ways, such as poor battery life, voice quality, and dropped calls. In 1G, the maximum achievable speed was 2.4 Kbps.

Second Generation (2G): In 2G, the first digital system was offered in 1991, providing improved mobile voice communication over 1G. In addition, Code-Division Multiple Access (CDMA) and Global System for Mobile (GSM) concepts were also discussed. In 2G, the maximum achievable speed was 1 Mpbs.

Third Generation (3G): When technology ventured from 2G GSM frameworks into 3G universal mobile telecommunication system (UMTS) framework, users encountered higher system speed and quicker download speed making constant video calls. 3G was the first mobile broadband system that was formed to provide the voice with some multimedia. The technology behind 3G was high-speed packet access (HSPA/HSPA+). 3G used MIMO for multiplying the power of the wireless network, and it also used packet switching for fast data transmission.

Fourth Generation (4G): It is purely mobile broadband standard. In digital mobile communication, it was observed information rate that upgraded from 20 to 60 Mbps in 4G [4]. It works on LTE and WiMAX technologies, as well as provides wider bandwidth up to 100 Mhz. It was launched in 2010.

Fourth Generation LTE-A (4.5G): It is an advanced version of standard 4G LTE. LTE-A uses MIMO technology to combine multiple antennas for both transmitters as well as a receiver. Using MIMO, multiple signals and multiple antennas can work simultaneously, making LTE-A three times faster than standard 4G. LTE-A offered an improved system limit, decreased deferral in the application server, access triple traffic (Data, Voice, and Video) wirelessly at any time anywhere in the world.LTE-A delivers speeds of over 42 Mbps and up to 90 Mbps.

Fifth Generation (5G): 5G is a pillar of digital transformation; it is a real improvement on all the previous mobile generation networks. 5G brings three different services for end user like Extreme mobile broadband (eMBB). It offers high-speed internet connectivity, greater bandwidth, moderate latency, UltraHD streaming videos, virtual reality and augmented reality (AR/VR) media, and many more. Massive machine type communication (eMTC), it provides long-range and broadband machine-type communication at a very cost-effective price with less power consumption. eMTC brings a high data rate service, low power, extended coverage via less device complexity through mobile carriers for IoT applications. Ultra-reliable low latency communication (URLLC) offers low-latency and ultra-high reliability, rich quality of service (QoS), which is not possible with traditional mobile network architecture. URLLC is designed for on-demand real-time interaction such as remote surgery, vehicle to vehicle (V2V) communication, industry 4.0, smart grids, intelligent transport system, etc. 5G faster than 4G and offers remote-controlled operation over a reliable network with zero delays. It provides down-link maximum throughput of up to 20 Gbps. In addition, 5G also supports 4G WWWW (4th Generation World Wide Wireless Web) [5] and is based on Internet protocol version 6 (IPv6) protocol. 5G provides unlimited internet connection at your convenience, anytime, anywhere with extremely high speed, high throughput, low-latency, higher reliability and scalability, and energy-efficient mobile communication technology [6]. 5G mainly divided in two parts 6 GHz 5G and Millimeter wave(mmWave) 5G.

6 GHz is a mid frequency band which works as a mid point between capacity and coverage to offer perfect environment for 5G connectivity. 6 GHz spectrum will provide high bandwidth with improved network performance. It offers continuous channels that will reduce the need for network densification when mid-band spectrum is not available and it makes 5G connectivity affordable at anytime, anywhere for everyone.

mmWave is an essential technology of 5G network which build high performance network. 5G mmWave offer diverse services that is why all network providers should add on this technology in their 5G deployment planning. There are lots of service providers who deployed 5G mmWave, and their simulation result shows that 5G mmwave is a far less used spectrum. It provides very high speed wireless communication and it also offers ultra-wide bandwidth for next generation mobile network.

The evolution of wireless mobile technologies are presented in Table 1. The abbreviations used in this paper are mentioned in Table 2.

### 1.2. Key Contributions

The objective of this survey is to provide a detailed guide of 5G key technologies, methods to researchers, and to help with understanding how the recent works addressed 5G problems and developed solutions to tackle the 5G challenges; i.e., what are new methods that must be applied and how can they solve problems? Highlights of the research article are as follows.

- This survey focused on the recent trends and development in the era of 5G and novel contributions by the researcher community and discussed technical details on essential aspects of the 5G advancement.
- In this paper, the evolution of the mobile network from 1G to 5G is presented. In addition, the growth of mobile communication under different attributes is also discussed.
- This paper covers the emerging applications and research groups working on 5G & different research areas in 5G wireless communication network with a descriptive taxonomy.
- This survey discusses the current vision of the 5G networks, advantages, applications, key technologies, and key features. Furthermore, machine learning prospects are also explored with the emerging requirements in the 5G era. The article also focused on technical aspects of 5G IoT Based approaches and optimization techniques for 5G.
- we provide an extensive overview and recent advancement of emerging technologies of 5G mobile network, namely, MIMO, Non-Orthogonal Multiple Access (NOMA), mmWave, Internet of Things (IoT), Machine Learning (ML), and optimization. Also, a technical summary is discussed by highlighting the context of current approaches and corresponding challenges.
- Security challenges and considerations while developing 5G technology are discussed.
- Finally, the paper concludes with the future directives.

The existing survey focused on architecture, key concepts, and implementation challenges and issues. In contrast, this survey covers the state-of-the-art techniques as well as corresponding recent novel developments by researchers. Various recent significant papers are discussed with the key technologies accelerating the development and production of 5G products.

## 2. Existing Surveys and Their Applicability

In this paper, a detailed survey on various technologies of 5G networks is presented. Various researchers have worked on different technologies of 5G networks. In this section, Table 3 gives a tabular representation of existing surveys of 5G networks. Massive MIMO, NOMA, small cell, mmWave, beamforming, and MEC are the six main pillars that helped to implement 5G networks in real life.

### 2.1. Limitations of Existing Surveys

**Figure 1:**
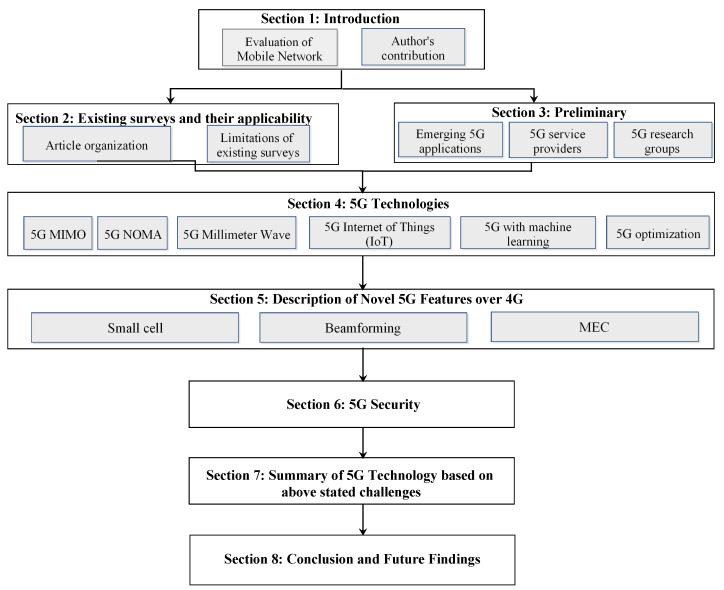
Systematic layout representation of survey.

The existing survey focused on architecture, key concepts, and implementation challenges and issues. The numerous current surveys focused on various 5G technologies with different parameters, and the authors did not cover all the technologies of the 5G network in detail with challenges and recent advancements. Few authors worked on MIMO (Non-Orthogonal Multiple Access) NOMA, MEC, small cell technologies. In contrast, some others worked on beamforming, Millimeter-wave (mmWave). But the existing survey did not cover all the technologies of the 5G network from a research and advancement perspective. No detailed survey is available in the market covering all the 5G network technologies and currently published research trade-offs. So, our main aim is to give a detailed study of all the technologies working on the 5G network. In contrast, this survey covers the state-of-the-art techniques as well as corresponding recent novel developments by researchers. Various recent significant papers are discussed with the key technologies accelerating the development and production of 5G products. This survey article collected key information about 5G technology and recent advancements, and it can be a kind of a guide for the reader. This survey provides an umbrella approach to bring multiple solutions and recent improvements in a single place to accelerate the 5G research with the latest key enabling solutions and reviews. A systematic layout representation of the survey in Figure 1. We provide a state-of-the-art comparative overview of the existing surveys on different technologies of 5G networks in Table 3.

### 2.2. Article Organization

This article is organized under the following sections. Section 2 presents existing surveys and their applicability. In Section 3, the preliminaries of 5G technology are presented. In Section 4, recent advances of 5G technology based on Massive MIMO, NOMA, Millimeter Wave, 5G with IoT, machine learning for 5G, and Optimization in 5G are provided. In Section 5, a description of novel 5G features over 4G is provided. Section 6 covered all the security concerns of the 5G network. Section 7, 5G technology based on above-stated challenges summarize in tabular form. Finally, Section 8 and Section 9 conclude the study, which paves the path for future research.

## 3. Preliminary Section

### 3.1. Emerging 5G Paradigms and Its Features

5G provides very high speed, low latency, and highly salable connectivity between multiple devices and IoT worldwide. 5G will provide a very flexible model to develop a modern generation of applications and industry goals [26,27]. There are many services offered by 5G network architecture are stated below:

Massive machine to machine communications: 5G offers novel, massive machine-to-machine communications [28], also known as the IoT [29], that provide connectivity between lots of machines without any involvement of humans. This service enhances the applications of 5G and provides connectivity between agriculture, construction, and industries [30].

Ultra-reliable low latency communications (URLLC): This service offers real-time management of machines, high-speed vehicle-to-vehicle connectivity, industrial connectivity and security principles, and highly secure transport system, and multiple autonomous actions. Low latency communications also clear up a different area where remote medical care, procedures, and operation are all achievable [31].

Enhanced mobile broadband: Enhance mobile broadband is an important use case of 5G system, which uses massive MIMO antenna, mmWave, beamforming techniques to offer very high-speed connectivity across a wide range of areas [32].

For communities: 5G provides a very flexible internet connection between lots of machines to make smart homes, smart schools, smart laboratories, safer and smart automobiles, and good health care centers [33].

For businesses and industry: As 5G works on higher spectrum ranges from 24 to 100 GHz. This higher frequency range provides secure low latency communication and high-speed wireless connectivity between IoT devices and industry 4.0, which opens a market for end-users to enhance their business models [34].

New and Emerging technologies: As 5G came up with many new technologies like beamforming, massive MIMO, mmWave, small cell, NOMA, MEC, and network slicing, it introduced many new features to the market. Like virtual reality (VR), users can experience the physical presence of people who are millions of kilometers away from them. Many new technologies like smart homes, smart workplaces, smart schools, smart sports academy also came into the market with this 5G Mobile network model [35].

### 3.2. Commercial Service Providers of 5G

5G provides high-speed internet browsing, streaming, and downloading with very high reliability and low latency. 5G network will change your working style, and it will increase new business opportunities and provide innovations that we cannot imagine. This section covers top service providers of 5G network [36,37].

**Ericsson:** Ericsson is a Swedish multinational networking and telecommunications company, investing around 25.62 billion USD in 5G network, which makes it the biggest telecommunication company. It claims that it is the only company working on all the continents to make the 5G network a global standard for the next generation wireless communication. Ericsson developed the first 5G radio prototype that enables the operators to set up the live field trials in their network, which helps operators understand how 5G reacts. It plays a vital role in the development of 5G hardware. It currently provides 5G services in over 27 countries with content providers like China Mobile, GCI, LGU+, AT&T, Rogers, and many more. It has 100 commercial agreements with different operators as of 2020.

**Verizon:** It is American multinational telecommunication which was founded in 1983. Verizon started offering 5G services in April 2020, and by December 2020, it has actively provided 5G services in 30 cities of the USA. They planned that by the end of 2021, they would deploy 5G in 30 more new cities. Verizon deployed a 5G network on mmWave, a very high band spectrum between 30 to 300 GHz. As it is a significantly less used spectrum, it provides very high-speed wireless communication. MmWave offers ultra-wide bandwidth for next-generation mobile networks. MmWave is a faster and high-band spectrum that has a limited range. Verizon planned to increase its number of 5G cells by 500% by 2020. Verizon also has an ultra wide-band flagship 5G service which is the best 5G service that increases the market price of Verizon.

**Nokia:** Nokia is a Finnish multinational telecommunications company which was founded in 1865. Nokia is one of the companies which adopted 5G technology very early. It is developing, researching, and building partnerships with various 5G renders to offer 5G communication as soon as possible. Nokia collaborated with Deutsche Telekom and Hamburg Port Authority and provided them 8000-hectare site for their 5G MoNArch project. Nokia is the only company that supplies 5G technology to all the operators of different countries like AT&T, Sprint, T-Mobile US and Verizon in the USA, Korea Telecom, LG U+ and SK Telecom in South Korea and NTT DOCOMO, KDDI, and SoftBank in Japan. Presently, Nokia has around 150+ agreements and 29 live networks all over the world. Nokia is continuously working hard on 5G technology to expand 5G networks all over the globe.

**AT&T:** AT&T is an American multinational company that was the first to deploy a 5G network in reality in 2018. They built a gigabit 5G network connection in Waco, TX, Kalamazoo, MI, and South Bend to achieve this. It is the first company that archives 1–2 gigabit per second speed in 2019. AT&T claims that it provides a 5G network connection among 225 million people worldwide by using a 6 GHz spectrum band.

**T-Mobile:** T-Mobile US (TMUS) is an American wireless network operator which was the first service provider that offers a real 5G nationwide network. The company knew that high-band 5G was not feasible nationwide, so they used a 600 MHz spectrum to build a significant portion of its 5G network. TMUS is planning that by 2024 they will double the total capacity and triple the full 5G capacity of T-Mobile and Sprint combined. The sprint buyout is helping T-Mobile move forward the company’s current market price to 129.98 USD.

**Samsung:** Samsung started their research in 5G technology in 2011. In 2013, Samsung successfully developed the world’s first adaptive array transceiver technology operating in the millimeter-wave Ka bands for cellular communications. Samsung provides several hundred times faster data transmission than standard 4G for core 5G mobile communication systems. The company achieved a lot of success in the next generation of technology, and it is considered one of the leading companies in the 5G domain.

**Qualcomm:** Qualcomm is an American multinational corporation in San Diego, California. It is also one of the leading company which is working on 5G chip. Qualcomm’s first 5G modem chip was announced in October 2016, and a prototype was demonstrated in October 2017. Qualcomm mainly focuses on building products while other companies talk about 5G; Qualcomm is building the technologies. According to one magazine, Qualcomm was working on three main areas of 5G networks. Firstly, radios that would use bandwidth from any network it has access to; secondly, creating more extensive ranges of spectrum by combining smaller pieces; and thirdly, a set of services for internet applications.

**ZTE Corporation:** ZTE Corporation was founded in 1985. It is a partially Chinese state-owned technology company that works in telecommunication. It was a leading company that worked on 4G LTE, and it is still maintaining its value and doing research and tests on 5G. It is the first company that proposed Pre5G technology with some series of solutions.

**NEC Corporation:** NEC Corporation is a Japanese multinational information technology and electronics corporation headquartered in Minato, Tokyo. ZTE also started their research on 5G, and they introduced a new business concept. NEC’s main aim is to develop 5G NR for the global mobile system and create secure and intelligent technologies to realize 5G services.

**Cisco:** Cisco is a USA networking hardware company that also sleeves up for 5G network. Cisco’s primary focus is to support 5G in three ways: Service—enable 5G services faster so all service providers can increase their business. Infrastructure—build 5G-oriented infrastructure to implement 5G more quickly. Automation—make a more scalable, flexible, and reliable 5G network. The companies know the importance of 5G, and they want to connect more than 30 billion devices in the next couple of years. Cisco intends to work on network hardening as it is a vital part of 5G network. Cisco used AI with deep learning to develop a 5G Security Architecture, enabling Secure Network Transformation.

### 3.3. 5G Research Groups

Many research groups from all over the world are working on a 5G wireless mobile network [38]. These groups are continuously working on various aspects of 5G. The list of those research groups are presented as follows: 5GNOW (5th Generation Non-Orthogonal Waveform for Asynchronous Signaling), NEWCOM (Network of Excellence in Wireless Communication), 5GIC (5G Innovation Center), NYU (New York University) Wireless, 5GPPP (5G Infrastructure Public-Private Partnership), EMPHATIC (Enhanced Multi-carrier Technology for Professional Adhoc and Cell-Based Communication), ETRI(Electronics and Telecommunication Research Institute), METIS (Mobile and wireless communication Enablers for the Twenty-twenty Information Society) [39]. The various research groups along with the research area are presented in Table 4.

### 3.4. 5G Applications

5G is faster than 4G and offers remote-controlled operation over a reliable network with zero delays. It provides down-link maximum throughput of up to 20 Gbps. In addition, 5G also supports 4G WWWW (4th Generation World Wide Wireless Web) [5] and is based on Internet protocol version 6 (IPv6) protocol. 5G provides unlimited internet connection at your convenience, anytime, anywhere with extremely high speed, high throughput, low-latency, higher reliability, greater scalablility, and energy-efficient mobile communication technology [6].

There are lots of applications of 5G mobile network are as follows:

1. **High-speed mobile network:** 5G is an advancement on all the previous mobile network technologies, which offers very high speed downloading speeds 0 of up to 10 to 20 Gbps. The 5G wireless network works as a fiber optic internet connection. 5G is different from all the conventional mobile transmission technologies, and it offers both voice and high-speed data connectivity efficiently. 5G offers very low latency communication of less than a millisecond, useful for autonomous driving and mission-critical applications. 5G will use millimeter waves for data transmission, providing higher bandwidth and a massive data rate than lower LTE bands. As 5 Gis a fast mobile network technology, it will enable virtual access to high processing power and secure and safe access to cloud services and enterprise applications. Small cell is one of the best features of 5G, which brings lots of advantages like high coverage, high-speed data transfer, power saving, easy and fast cloud access, etc. [40].
2. **Entertainment and multimedia:** In one analysis in 2015, it was found that more than 50 percent of mobile internet traffic was used for video downloading. This trend will surely increase in the future, which will make video streaming more common. 5G will offer High-speed streaming of 4K videos with crystal clear audio, and it will make a high definition virtual world on your mobile. 5G will benefit the entertainment industry as it offers 120 frames per second with high resolution and higher dynamic range video streaming, and HD TV channels can also be accessed on mobile devices without any interruptions. 5G provides low latency high definition communication so augmented reality (AR), and virtual reality (VR) will be very easily implemented in the future. Virtual reality games are trendy these days, and many companies are investing in HD virtual reality games. The 5G network will offer high-speed internet connectivity with a better gaming experience [41].
3. **Internet of Things**—connecting everything: the 5G mobile network plays a significant role in developing the Internet of Things (IoT). IoT will connect many things with the internet like appliances, sensors, devices, objects, and applications. These applications will collect lots of data from different devices and sensors. 5G will provide very high-speed internet connectivity for data collection, transmission, control, and processing. 5G is a flexible network with unused spectrum availability, and it offers very low-cost deployment that is why it is the most efficient technology for IoT [42]. In many areas, 5G provide benefit to IoT are as follows:
  - **Smart homes**: smart home appliances and products are in demand these days. The 5G network makes smart homes more real as it offers high-speed connectivity and monitoring of smart appliances. Smart home appliances are easily accessed and configured from remote locations using the 5G network as it offers very high-speed low latency communication.
  - **Smart cities:** 5G wireless network also helps develop smart cities applications such as automatic traffic management, weather update, local area broadcasting, energy-saving, efficient power supply, smart lighting system, water resource management, crowd management, emergency control, etc.
  - **Industrial IoT:** 5G wireless technology will provide lots of features for future industries such as safety, process tracking, smart packing, shipping, energy efficiency, automation of equipment, predictive maintenance, and logistics. 5G smart sensor technology also offers smarter, safer, cost-effective, and energy-saving industrial IoT operations.
  - **Smart Farming:** 5G technology will play a crucial role in agriculture and smart farming. 5G sensors and GPS technology will help farmers track live attacks on crops and manage them quickly. These smart sensors can also be used for irrigation, pest, insect, and electricity control.
  - **Autonomous Driving:** The 5G wireless network offers very low latency high-speed communication, significant for autonomous driving. It means self-driving cars will come to real life soon with 5G wireless networks. Using 5G autonomous cars can easily communicate with smart traffic signs, objects, and other vehicles running on the road. 5G’s low latency feature makes self-driving more real as every millisecond is essential for autonomous vehicles, decision-making is done in microseconds to avoid accidents.
4. **Healthcare and mission-critical applications:** 5G technology will bring modernization in medicine where doctors and practitioners can perform advanced medical procedures. The 5G network will provide connectivity between all classrooms, so attending seminars and lectures will be easier. Through 5G technology, patients can connect with doctors and take their advice. Scientists are building smart medical devices which can help people with chronic medical conditions. The 5G network will boost the healthcare industry with smart devices, the internet of medical things, smart sensors, HD medical imaging technologies, and smart analytics systems. 5G will help access cloud storage, so accessing healthcare data will be very easy from any location worldwide. Doctors and medical practitioners can easily store and share large files like MRI reports within seconds using the 5G network.
5. **Satellite Internet:** In many remote areas, ground base stations are not available, so 5G will play a crucial role in providing connectivity in such areas. The 5G network will provide connectivity using satellite systems, and the satellite system uses a constellation of multiple small satellites to provide connectivity in urban and rural areas across the world.

## 4. 5G Technologies

This section describes recent advances of 5G Massive MIMO, 5G NOMA, 5G millimeter wave, 5G IOT, 5G with machine learning, and 5G optimization-based approaches. In addition, the summary is also presented in each subsection that paves the researchers for the future research direction.

### 4.1. 5G Massive MIMO

Multiple-input-multiple-out (MIMO) is a very important technology for wireless systems. It is used for sending and receiving multiple signals simultaneously over the same radio channel. MIMO plays a very big role in WI-FI, 3G, 4G, and 4G LTE-A networks. MIMO is mainly used to achieve high spectral efficiency and energy efficiency but it was not up to the mark MIMO provides low throughput and very low reliable connectivity. To resolve this, lots of MIMO technology like single user MIMO (SU-MIMO), multiuser MIMO (MU-MIMO) and network MIMO were used. However, these new MIMO also did not still fulfill the demand of end users. Massive MIMO is an advancement of MIMO technology used in the 5G network in which hundreds and thousands of antennas are attached with base stations to increase throughput and spectral efficiency. Multiple transmit and receive antennas are used in massive MIMO to increase the transmission rate and spectral efficiency. When multiple UEs generate downlink traffic simultaneously, massive MIMO gains higher capacity. Massive MIMO uses extra antennas to move energy into smaller regions of space to increase spectral efficiency and throughput [43]. In traditional systems data collection from smart sensors is a complex task as it increases latency, reduced data rate and reduced reliability. While massive MIMO with beamforming and huge multiplexing techniques can sense data from different sensors with low latency, high data rate and higher reliability. Massive MIMO will help in transmitting the data in real-time collected from different sensors to central monitoring locations for smart sensor applications like self-driving cars, healthcare centers, smart grids, smart cities, smart highways, smart homes, and smart enterprises [44].

**Figure 2:**
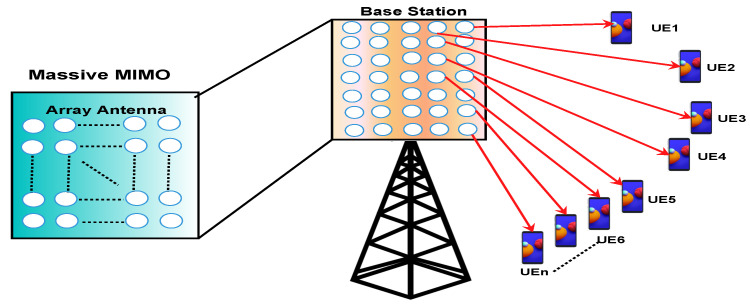
Pictorial representation of multi-input and multi-output (MIMO).

Highlights of 5G Massive MIMO technology are as follows:

1. **Data rate:** Massive MIMO is advised as the one of the dominant technologies to provide wireless high speed and high data rate in the gigabits per seconds.
2. **The relationship between wave frequency and antenna size:** Both are inversely proportional to each other. It means lower frequency signals need a bigger antenna and vise versa.
3. **Number of user:** From 1G to 4G technology one cell consists of 10 antennas. But, in 5G technologies one cell consist of more than 100 antennas. Hence, one small cell at the same time can handle multiple users [45]. As shown in Figure 2.
4. **MIMO role in 5G:** Massive MIMO will play a crucial role in the deployment of future 5G mobile communication as greater spectral and energy efficiency could be enabled.

#### State-of-the-Art Approaches

Plenty of approaches were proposed to resolve the issues of conventional MIMO [7].

The MIMO multirate, feed-forward controller is suggested by Mae et al. [46]. In the simulation, the proposed model generates the smooth control input, unlike the conventional MIMO, which generates oscillated control inputs. It also outperformed concerning the error rate. However, a combination of multirate and single rate can be used for better results.

The performance of stand-alone MIMO, distributed MIMO with and without corporation MIMO, was investigated by Panzner et al. [47]. In addition, an idea about the integration of large scale in the 5G technology was also presented. In the experimental analysis, different MIMO configurations are considered. The variation in the ratio of overall transmit antennas to spatial is deemed step-wise from equality to ten.

The simulation of massive MIMO noncooperative and cooperative systems for down-link behavior was performed by He et al. [48]. It depends on present LTE systems, which deal with various antennas in the base station set-up. It was observed that collaboration in different BS improves the system behaviors, whereas throughput is reduced slightly in this approach. However, a new method can be developed which can enhance both system behavior and throughput.

In [8], different approaches that increased the energy efficiency benefits provided by massive MIMO were presented. They analyzed the massive MIMO technology and described the detailed design of the energy consumption model for massive MIMO systems. This article has explored several techniques to enhance massive MIMO systems’ energy efficiency (EE) gains. This paper reviews standard EE-maximization approaches for the conventional massive MIMO systems, namely, scaling number of antennas, real-time implementing low-complexity operations at the base station (BS), power amplifier losses minimization, and radio frequency (RF) chain minimization requirements. In addition, open research direction is also identified.

In [49], various existing approaches based on different antenna selection and scheduling, user selection and scheduling, and joint antenna and user scheduling methods adopted in massive MIMO systems are presented in this paper. The objective of this survey article was to make awareness about the current research and future research direction in MIMO for systems. They analyzed that complete utilization of resources and bandwidth was the most crucial factor which enhances the sum rate.

In [50], authors discussed the development of various techniques for pilot contamination. To calculate the impact of pilot contamination in time division duplex (TDD) massive MIMO system, TDD and frequency division duplexing FDD patterns in massive MIMO techniques are used. They discussed different issues in pilot contamination in TDD massive MIMO systems with all the possible future directions of research. They also classified various techniques to generate the channel information for both pilot-based and subspace-based approaches.

In [19], the authors defined the uplink and downlink services for a massive MIMO system. In addition, it maintains a performance matrix that measures the impact of pilot contamination on different performances. They also examined the various application of massive MIMO such as small cells, orthogonal frequency-division multiplexing (OFDM) schemes, massive MIMO IEEE 802, 3rd generation partnership project (3GPP) specifications, and higher frequency bands. They considered their research work crucial for cutting edge massive MIMO and covered many issues like system throughput performance and channel state acquisition at higher frequencies.

In [13], various approaches were suggested for MIMO future generation wireless communication. They made a comparative study based on performance indicators such as peak data rate, energy efficiency, latency, throughput, etc. The key findings of this survey are as follows: (1) spatial multiplexing improves the energy efficiency; (2) design of MIMO play a vital role in the enhancement of throughput; (3) enhancement of mMIMO focusing on energy & spectral performance; (4) discussed the future challenges to improve the system design.

In [51], the study of large-scale MIMO systems for an energy-efficient system sharing method was presented. For the resource allocation, circuit energy and transmit energy expenditures were taken into consideration. In addition, the optimization techniques were applied for an energy-efficient resource sharing system to enlarge the energy efficiency for individual QoS and energy constraints. The author also examined the BS configuration, which includes homogeneous and heterogeneous UEs. While simulating, they discussed that the total number of transmit antennas plays a vital role in boosting energy efficiency. They highlighted that the highest energy efficiency was obtained when the BS was set up with 100 antennas that serve 20 UEs.

This section includes various works done on 5G MIMO technology by different author’s. Table 5 shows how different author’s worked on improvement of various parameters such as throughput, latency, energy efficiency, and spectral efficiency with 5G MIMO technology.

### 4.2. 5G Non-Orthogonal Multiple Access (NOMA)

NOMA is a very important radio access technology used in next generation wireless communication. Compared to previous orthogonal multiple access techniques, NOMA offers lots of benefits like high spectrum efficiency, low latency with high reliability and high speed massive connectivity. NOMA mainly works on a baseline to serve multiple users with the same resources in terms of time, space and frequency. NOMA is mainly divided into two main categories one is code domain NOMA and another is power domain NOMA. Code-domain NOMA can improve the spectral efficiency of mMIMO, which improves the connectivity in 5G wireless communication. Code-domain NOMA was divided into some more multiple access techniques like sparse code multiple access, lattice-partition multiple access, multi-user shared access and pattern-division multiple access [52]. Power-domain NOMA is widely used in 5G wireless networks as it performs well with various wireless communication techniques such as MIMO, beamforming, space-time coding, network coding, full-duplex and cooperative communication etc. [53]. The conventional orthogonal frequency-division multiple access (OFDMA) used by 3GPP in 4G LTE network provides very low spectral efficiency when bandwidth resources are allocated to users with low channel state information (CSI). NOMA resolved this issue as it enables users to access all the subcarrier channels so bandwidth resources allocated to the users with low CSI can still be accessed by the users with strong CSI which increases the spectral efficiency. The 5G network will support heterogeneous architecture in which small cell and macro base stations work for spectrum sharing. NOMA is a key technology of the 5G wireless system which is very helpful for heterogeneous networks as multiple users can share their data in a small cell using the NOMA principle.The NOMA is helpful in various applications like ultra-dense networks (UDN), machine to machine (M2M) communication and massive machine type communication (mMTC). As NOMA provides lots of features it has some challenges too such as NOMA needs huge computational power for a large number of users at high data rates to run the SIC algorithms. Second, when users are moving from the networks, to manage power allocation optimization is a challenging task for NOMA [54]. Hybrid NOMA (HNOMA) is a combination of power-domain and code-domain NOMA. HNOMA uses both power differences and orthogonal resources for transmission among multiple users. As HNOMA is using both power-domain NOMA and code-domain NOMA it can achieve higher spectral efficiency than Power-domain NOMA and code-domain NOMA. In HNOMA multiple groups can simultaneously transmit signals at the same time. It uses a message passing algorithm (MPA) and successive interference cancellation (SIC)-based detection at the base station for these groups [55].

**Figure 3:**
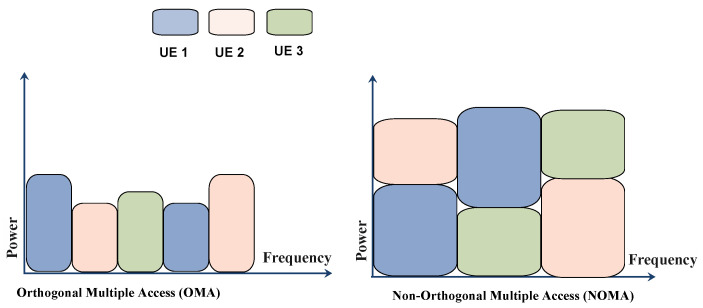
Pictorial representation of orthogonal and Non-Orthogonal Multiple Access (NOMA).

Highlights of 5G NOMA technology as follows:

- NOMA is different than all the previous orthogonal access techniques such as TDMA, FDMA and CDMA. In NOMA, multiple users work simultaneously in the same band with different power levels. As shown in Figure 3.
- NOMA provides higher data rates and resolves all the loop holes of OMA that makes 5G mobile network more scalable and reliable.
- As multiple users use same frequency band simultaneously it increases the performance of whole network.
- To setup intracell and intercell interference NOMA provides nonorthogonal transmission on the transmitter end.
- The primary fundamental of NOMA is to improve the spectrum efficiency by strengthening the ramification of receiver.

#### State-of-the-Art of Approaches

A plenty of approaches were developed to address the various issues in NOMA.

A novel approach to address the multiple receiving signals at the same frequency is proposed in [22]. In NOMA, multiple users use the same sub-carrier, which improves the fairness and throughput of the system. As a nonorthogonal method is used among multiple users, at the time of retrieving the user’s signal at the receiver’s end, joint processing is required. They proposed solutions to optimize the receiver and the radio resource allocation of uplink NOMA. Firstly, the authors proposed an iterative MUDD which utilizes the information produced by the channel decoder to improve the performance of the multiuser detector. After that, the author suggested a power allocation and novel subcarrier that enhances the users’ weighted sum rate for the NOMA scheme. Their proposed model showed that NOMA performed well as compared to OFDM in terms of fairness and efficiency.

In [53], the author’s reviewed a power-domain NOMA that uses superposition coding (SC) and successive interference cancellation (SIC) at the transmitter and the receiver end. Lots of analyses were held that described that NOMA effectively satisfies user data rate demands and network-level of 5G technologies. The paper presented a complete review of recent advances in the 5G NOMA system. It showed the comparative analysis regarding allocation procedures, user fairness, state-of-the-art efficiency evaluation, user pairing pattern, etc. The study also analyzes NOMA’s behavior when working with other wireless communication techniques, namely, beamforming, MIMO, cooperative connections, network, space-time coding, etc.

In [9], the authors proposed NOMA with MEC, which improves the QoS as well as reduces the latency of the 5G wireless network. This model increases the uplink NOMA by decreasing the user’s uplink energy consumption. They formulated an optimized NOMA framework that reduces the energy consumption of MEC by using computing and communication resource allocation, user clustering, and transmit powers.

In [10], the authors proposed a model which investigates outage probability under average channel state information CSI and data rate in full CSI to resolve the problem of optimal power allocation, which increase the NOMA downlink system among users. They developed simple low-complexity algorithms to provide the optimal solution. The obtained simulation results showed NOMA’s efficiency, achieving higher performance fairness compared to the TDMA configurations. It was observed from the results that NOMA, through the appropriate power amplifiers (PA), ensures the high-performance fairness requirement for the future 5G wireless communication networks.

In [56], researchers discussed that the NOMA technology and waveform modulation techniques had been used in the 5G mobile network. Therefore, this research gave a detailed survey of non-orthogonal waveform modulation techniques and NOMA schemes for next-generation mobile networks. By analyzing and comparing multiple access technologies, they considered the future evolution of these technologies for 5G mobile communication.

In [57], the authors surveyed non-orthogonal multiple access (NOMA) from the development phase to the recent developments. They have also compared NOMA techniques with traditional OMA techniques concerning information theory. The author discussed the NOMA schemes categorically as power and code domain, including the design principles, operating principles, and features. Comparison is based upon the system’s performance, spectral efficiency, and the receiver’s complexity. Also discussed are the future challenges, open issues, and their expectations of NOMA and how it will support the key requirements of 5G mobile communication systems with massive connectivity and low latency.

In [17], authors present the first review of an elementary NOMA model with two users, which clarify its central precepts. After that, a general design with multicarrier supports with a random number of users on each sub-carrier is analyzed. In performance evaluation with the existing approaches, resource sharing and multiple-input multiple-output NOMA are examined. Furthermore, they took the key elements of NOMA and its potential research demands. Finally, they reviewed the two-user SC-NOMA design and a multi-user MC-NOMA design to highlight NOMA’s basic approaches and conventions. They also present the research study about the performance examination, resource assignment, and MIMO in NOMA.

In this section, various works by different authors done on 5G NOMA technology is covered. Table 6 shows how other authors worked on the improvement of various parameters such as spectral efficiency, fairness, and computing capacity with 5G NOMA technology.

### 4.3. 5G Millimeter Wave (mmWave)

Millimeter wave is an extremely high frequency band, which is very useful for 5G wireless networks. MmWave uses 30 GHz to 300 GHz spectrum band for transmission. The frequency band between 30 GHz to 300 GHz is known as mmWave because these waves have wavelengths between 1 to 10 mm. Till now radar systems and satellites are only using mmWave as these are very fast frequency bands which provide very high speed wireless communication. Many mobile network providers also started mmWave for transmitting data between base stations. Using two ways the speed of data transmission can be improved one is by increasing spectrum utilization and second is by increasing spectrum bandwidth. Out of these two approaches increasing bandwidth is quite easy and better. The frequency band below 5 GHz is very crowded as many technologies are using it so to boost up the data transmission rate 5G wireless network uses mmWave technology which instead of increasing spectrum utilization, increases the spectrum bandwidth [58]. To maximize the signal bandwidth in wireless communication the carrier frequency should also be increased by 5% because the signal bandwidth is directly proportional to carrier frequencies. The frequency band between 28 GHz to 60 GHz is very useful for 5G wireless communication as 28 GHz frequency band offers up to 1 GHz spectrum bandwidth and 60 GHz frequency band offers 2 GHz spectrum bandwidth. 4G LTE provides 2 GHz carrier frequency which offers only 100 MHz spectrum bandwidth. However, the use of mmWave increases the spectrum bandwidth 10 times, which leads to better transmission speeds [59,60].

**Figure 4:**
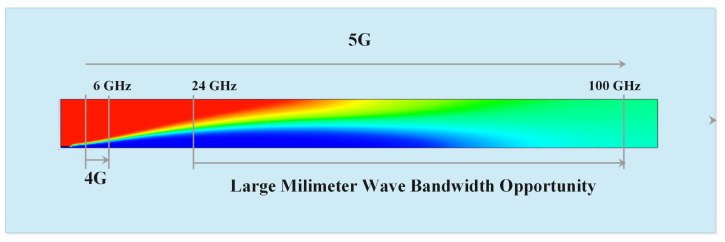
Pictorial representation of millimeter wave.

Highlights of 5G mmWave are as follows:

- In the technological world, everyone uses WiMax, GPS, wifi, 4G, 3G, L-Band, S-Band, C- Band Satellite, etc., for communication. The radio frequency spectrum of these technologies is minimal, which lies between 1 GHz to 6 GHz. Hence, it is very crowded. The spectrum range from 30 GHz to 300 GHz, known as mmWave, is less utilized and still not allocated to other communication technologies. After a long time, the range from 24 GHz to 100 GHz is allocated to 5G. As shown in Figure 4.
- The 5G mmWave offer three advantages: (1) MmWave is very less used new Band, (2) MmWave signals carry more data than lower frequency wave, and (3) MmWave can be incorporated with MIMO antenna with the potential to offer a higher magnitude capacity compared to current communication systems.

#### State-of-the-Art of Approaches

In [11], the authors presented the survey of mmWave communications for 5G. The advantage of mmWave communications is adaptability, i.e., it supports the architectures and protocols up-gradation, which consists of integrated circuits, systems, etc. The authors over-viewed the present solutions and examined them concerning effectiveness, performance, and complexity. They also discussed the open research issues of mmWave communications in 5G concerning the software-defined network (SDN) architecture, network state information, efficient regulation techniques, and the heterogeneous system.

In [61], the authors present the recent work done by investigators in 5G; they discussed the design issues and demands of mmWave 5G antennas for cellular handsets. After that, they designed a small size and low-profile 60 GHz array of antenna units that contain 3D planer mesh-grid antenna elements. For the future prospect, a framework is designed in which antenna components are used to operate cellular handsets on mmWave 5G smartphones. In addition, they cross-checked the mesh-grid array of antennas with the polarized beam for upcoming hardware challenges.

In [12], the authors considered the suitability of the mmWave band for 5G cellular systems. They suggested a resource allocation system for concurrent D2D communications in mmWave 5G cellular systems, and it improves network efficiency and maintains network connectivity. This research article can serve as guidance for simulating D2D communications in mmWave 5G cellular systems. Massive mmWave BS may be set up to obtain a high delivery rate and aggregate efficiency. Therefore, many wireless users can hand off frequently between the mmWave base terminals, and it emerges the demand to search the neighbor having better network connectivity.

In [62], the authors provided a brief description of the cellular spectrum which ranges from 1 GHz to 3 GHz and is very crowed. In addition, they presented various noteworthy factors to set up mmWave communications in 5G, namely, channel characteristics regarding mmWave signal attenuation due to free space propagation, atmospheric gaseous, and rain. In addition, hybrid beamforming architecture in the mmWave technique is analyzed. They also suggested methods for the blockage effect in mmWave communications due to penetration damage. Finally, the authors have studied designing the mmWave transmission with small beams in nonorthogonal device-to-device communication.

This section covered various works done on 5G mmWave technology. The Table 7 shows how different author’s worked on the improvement of various parameters i.e., transmission rate, coverage, and cost, with 5G mmWave technology.

### 4.4. 5G IoT Based Approaches

The 5G mobile network plays a big role in developing the Internet of Things (IoT). IoT will connect lots of things with the internet like appliances, sensors, devices, objects, and applications. These applications will collect lots of data from different devices and sensors. 5G will provide very high speed internet connectivity for data collection, transmission, control, and processing. 5G is a flexible network with unused spectrum availability and it offers very low cost deployment that is why it is the most efficient technology for IoT [63]. In many areas, 5G provides benefits to IoT, and below are some examples:

Smart homes: smart home appliances and products are in demand these days. The 5G network makes smart homes more real as it offers high speed connectivity and monitoring of smart appliances. Smart home appliances are easily accessed and configured from remote locations using the 5G network, as it offers very high speed low latency communication.

Smart cities: 5G wireless network also helps in developing smart cities applications such as automatic traffic management, weather update, local area broadcasting, energy saving, efficient power supply, smart lighting system, water resource management, crowd management, emergency control, etc.

Industrial IoT: 5G wireless technology will provide lots of features for future industries such as safety, process tracking, smart packing, shipping, energy efficiency, automation of equipment, predictive maintenance and logistics. 5G smart sensor technology also offers smarter, safer, cost effective, and energy-saving industrial operation for industrial IoT.

Smart Farming: 5G technology will play a crucial role for agriculture and smart farming. 5G sensors and GPS technology will help farmers to track live attacks on crops and manage them quickly. These smart sensors can also be used for irrigation control, pest control, insect control, and electricity control.

Autonomous Driving: 5G wireless network offers very low latency high speed communication which is very significant for autonomous driving. It means self-driving cars will come to real life soon with 5G wireless networks. Using 5G autonomous cars can easily communicate with smart traffic signs, objects and other vehicles running on the road. 5G’s low latency feature makes self-driving more real as every millisecond is important for autonomous vehicles, decision taking is performed in microseconds to avoid accidents [64].

**Figure 5:**
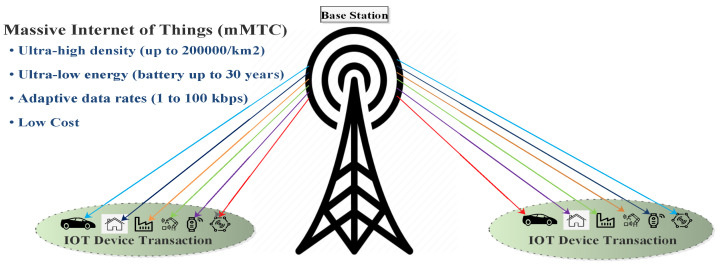
Pictorial representation of IoT with 5G.

Highlights of 5G IoT are as follows:

- IoT is termed as “Internet of Things.” It provides machine-to-machine (M2M) communication and shares information between heterogeneous devices without human interference. As shown in the Figure 5.
- 5G with IoT is a new feature of next-generation mobile communication, which provides a high-speed internet connection between moderated devices. 5G IoT also offers smart homes, smart devices, sensors, smart transportation systems, smart industries, etc., for end-users to make them smarter.
- IoT deals with moderate devices which connect through the internet. The approach of the IoT has made the consideration of the research associated with the outcome of providing wearable, smart-phones, sensors, smart transportation systems, smart devices, washing machines, tablets, etc., and these diverse systems are associated to a common interface with the intelligence to connect.
- Significant IoT applications include private healthcare systems, traffic management, industrial management, and tactile internet, etc.

#### State-of-the-Art of Approaches

Plenty of approaches is devised to address the issues of IoT [14,65,66].

In [65], the paper focuses on 5G mobile systems due to the emerging trends and developing technologies, which results in the exponential traffic growth in IoT. The author surveyed the challenges and demands during deployment of the massive IoT applications with the main focus on mobile networking. The author reviewed the features of standard IoT infrastructure, along with the cellular-based, low-power wide-area technologies (LPWA) such as eMTC, extended coverage (EC)-GSM-IoT, as well as noncellular, low-power wide-area (LPWA) technologies such as SigFox, LoRa etc.

In [14], the authors presented how 5G technology copes with the various issues of IoT today. It provides a brief review of existing and forming 5G architectures. The survey indicates the role of 5G in the foundation of the IoT ecosystem. IoT and 5G can easily combine with improved wireless technologies to set up the same ecosystem that can fulfill the current requirement for IoT devices. 5G can alter nature and will help to expand the development of IoT devices. As the process of 5G unfolds, global associations will find essentials for setting up a cross-industry engagement in determining and enlarging the 5G system.

In [66], the author introduced an IoT authentication scheme in a 5G network, with more excellent reliability and dynamic. The scheme proposed a privacy-protected procedure for selecting slices; it provided an additional fog node for proper data transmission and service types of the subscribers, along with service-oriented authentication and key understanding to maintain the secrecy, precision of users, and confidentiality of service factors. Users anonymously identify the IoT servers and develop a vital channel for service accessibility and data cached on local fog nodes and remote IoT servers. The author performed a simulation to manifest the security and privacy preservation of the user over the network.

This section covered various works done on 5G IoT by multiple authors. Table 8 shows how different author’s worked on the improvement of numerous parameters, i.e., data rate, security requirement, and performance with 5G IoT.

### 4.5. Machine Learning Techniques for 5G

Various machine learning (ML) techniques were applied in 5G networks and mobile communication. It provides a solution to multiple complex problems, which requires a lot of hand-tuning. ML techniques can be broadly classified as supervised, unsupervised, and reinforcement learning. Let’s discuss each learning technique separately and where it impacts the 5G network.

Supervised Learning, where user works with labeled data; some 5G network problems can be further categorized as classification and regression problems. Some regression problems such as scheduling nodes in 5G and energy availability can be predicted using Linear Regression (LR) algorithm. To accurately predict the bandwidth and frequency allocation Statistical Logistic Regression (SLR) is applied. Some supervised classifiers are applied to predict the network demand and allocate network resources based on the connectivity performance; it signifies the topology setup and bit rates. Support Vector Machine (SVM) and NN-based approximation algorithms are used for channel learning based on observable channel state information. Deep Neural Network (DNN) is also employed to extract solutions for predicting beamforming vectors at the BS’s by taking mapping functions and uplink pilot signals into considerations.

In unsupervised Learning, where the user works with unlabeled data, various clustering techniques are applied to enhance network performance and connectivity without interruptions. K-means clustering reduces the data travel by storing data centers content into clusters. It optimizes the handover estimation based on mobility pattern and selection of relay nodes in the V2V network. Hierarchical clustering reduces network failure by detecting the intrusion in the mobile wireless network; unsupervised soft clustering helps in reducing latency by clustering fog nodes. The nonparametric Bayesian unsupervised learning technique reduces traffic in the network by actively serving the user’s requests and demands. Other unsupervised learning techniques such as Adversarial Auto Encoders (AAE) and Affinity Propagation Clustering techniques detect irregular behavior in the wireless spectrum and manage resources for ultradense small cells, respectively.

In case of an uncertain environment in the 5G wireless network, reinforcement learning (RL) techniques are employed to solve some problems. Actor-critic reinforcement learning is used for user scheduling and resource allocation in the network. Markov decision process (MDP) and Partially Observable MDP (POMDP) is used for Quality of Experience (QoE)-based handover decision-making for Hetnets. Controls packet call admission in HetNets and channel access process for secondary users in a Cognitive Radio Network (CRN). Deep RL is applied to decide the communication channel and mobility and speeds up the secondary user’s learning rate using an antijamming strategy. Deep RL is employed in various 5G network application parameters such as resource allocation and security [67]. Table 9 shows the state-of-the-art ML-based solution for 5G network.

**Figure 6:**
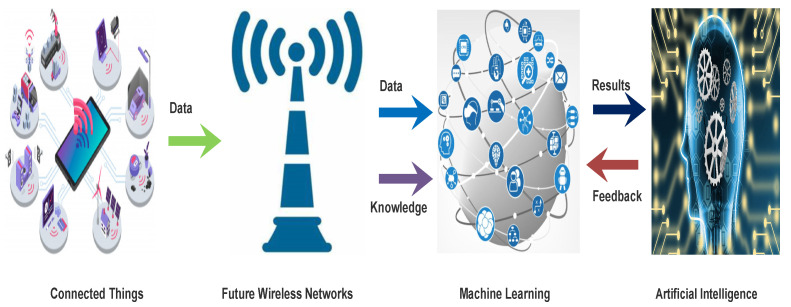
Pictorial representation of machine learning (ML) in 5G.

Highlights of machine learning techniques for 5G are as follows:

- Machine learning (ML) is a part of artificial intelligence. It processes and analyses the data that automates a systematic model that finds patterns and carries out decisions with minimum human interference. As shown in the Figure 6.
- In ML, a model will be defined which fulfills the desired requirements through which desired results are obtained. In the later stage, it examines accuracy from obtained results.
- ML plays a vital role in 5G network analysis for threat detection, network load prediction, final arrangement, and network formation. Searching for a better balance between power, length of antennas, area, and network thickness crossed with the spontaneous use of services in the universe of individual users and types of devices.

#### State-of-the-Art of Approaches

In [79], author’s firstly describes the demands for the traditional authentication procedures and benefits of intelligent authentication. The intelligent authentication method was established to improve security practice in 5G-and-beyond wireless communication systems. Thereafter, the machine learning paradigms for intelligent authentication were organized into parametric and non-parametric research methods, as well as supervised, unsupervised, and reinforcement learning approaches. As a outcome, machine learning techniques provide a new paradigm into authentication under diverse network conditions and unstable dynamics. In addition, prompt intelligence to the security management to obtain cost-effective, better reliable, model-free, continuous, and situation-aware authentication.

In [68], the authors proposed a machine learning-based model to predict the traffic load at a particular location. They used a mobile network traffic dataset to train a model that can calculate the total number of user requests at a time. To launch access and mobility management function (AMF) instances according to the requirement as there were no predictions of user request the performance automatically degrade as AMF does not handle these requests at a time. Earlier threshold-based techniques were used to predict the traffic load, but that approach took too much time; therefore, the authors proposed RNN algorithm-based ML to predict the traffic load, which gives efficient results.

In [15], authors discussed the issue of network slice admission, resource allocation among subscribers, and how to maximize the profit of infrastructure providers. The author proposed a network slice admission control algorithm based on SMDP (decision-making process) that guarantees the subscribers’ best acceptance policies and satisfiability (tenants). They also suggested novel N3AC, a neural network-based algorithm that optimizes performance under various configurations, significantly outperforms practical and straightforward approaches.

This section includes various works done on 5G ML by different authors. Table 10 shows the state-of-the-art work on the improvement of various parameters such as energy efficiency, Quality of Services (QoS), and latency with 5G ML.

### 4.6. Optimization Techniques for 5G

Optimization techniques may be applied to capture NP-Complete or NP-Hard problems in 5G technology. This section briefly describes various research works suggested for 5G technology based on optimization techniques.

In [80], Massive MIMO technology is used in 5G mobile network to make it more flexible and scalable. The MIMO implementation in 5G needs a significant number of radio frequencies is required in the RF circuit that increases the cost and energy consumption of the 5G network. This paper provides a solution that increases the cost efficiency and energy efficiency with many radio frequency chains for a 5G wireless communication network. They give an optimized energy efficient technique for MIMO antenna and mmWave technologies based 5G mobile communication network. The proposed Energy Efficient Hybrid Precoding (EEHP) algorithm to increase the energy efficiency for the 5G wireless network. This algorithm minimizes the cost of an RF circuit with a large number of RF chains.

In [16], authors have discussed the growing demand for energy efficiency in the next-generation networks. In the last decade, they have figured out the things in wireless transmissions, which proved a change towards pursuing green communication for the next generation system. The importance of adopting the correct EE metric was also reviewed. Further, they worked through the different approaches that can be applied in the future for increasing the network’s energy and posed a summary of the work that was completed previously to enhance the energy productivity of the network using these capabilities. A system design for EE development using relay selection was also characterized, along with an observation of distinct algorithms applied for EE in relay-based ecosystems.

In [81], authors presented how AI-based approach is used to the setup of Self Organizing Network (SON) functionalities for radio access network (RAN) design and optimization. They used a machine learning approach to predict the results for 5G SON functionalities. Firstly, the input was taken from various sources; then, prediction and clustering-based machine learning models were applied to produce the results. Multiple AI-based devices were used to extract the knowledge analysis to execute SON functionalities smoothly. Based on results, they tested how self-optimization, self-testing, and self-designing are done for SON. The author also describes how the proposed mechanism classifies in different orders.

In [82], investigators examined the working of OFDM in various channel environments. They also figured out the changes in frame duration of the 5G TDD frame design. Subcarrier spacing is beneficial to obtain a small frame length with control overhead. They provided various techniques to reduce the growing guard period (GP) and cyclic prefix (CP) like complete utilization of multiple subcarrier spacing, management and data parts of frame at receiver end, various uses of timing advance (TA) or total control of flexible CP size.

This section includes various works that were done on 5G optimization by different authors. Table 11 shows how other authors worked on the improvement of multiple parameters such as energy efficiency, power optimization, and latency with 5G optimization.

## 5. Description of Novel 5G Features over 4G

This section presents descriptions of various novel features of 5G, namely, the concept of small cell, beamforming, and MEC.

### 5.1. Small Cell

Small cells are low-powered cellular radio access nodes which work in the range of 10 meters to a few kilometers. Small cells play a very important role in implementation of the 5G wireless network. Small cells are low power base stations which cover small areas. Small cells are quite similar with all the previous cells used in various wireless networks. However, these cells have some advantages like they can work with low power and they are also capable of working with high data rates. Small cells help in rollout of 5G network with ultra high speed and low latency communication. Small cells in the 5G network use some new technologies like MIMO, beamforming, and mmWave for high speed data transmission. The design of small cells hardware is very simple so its implementation is quite easier and faster. There are three types of small cell tower available in the market. Femtocells, picocells, and microcells [83]. As shown in the Table 12.

**Figure 7:**
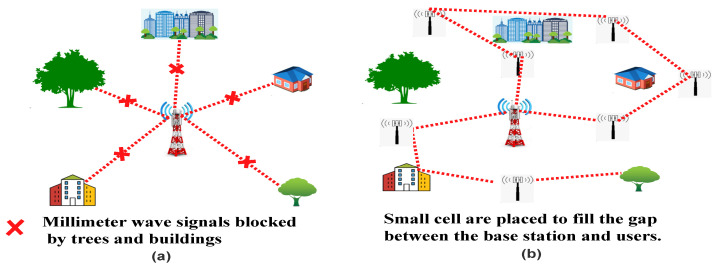
Pictorial representation of communication with and without small cells.

MmWave is a very high band spectrum between 30 to 300 GHz. As it is a significantly less used spectrum, it provides very high-speed wireless communication. MmWave offers ultra-wide bandwidth for next-generation mobile networks. MmWave has lots of advantages, but it has some disadvantages, too, such as mmWave signals are very high-frequency signals, so they have more collision with obstacles in the air which cause the signals loses energy quickly. Buildings and trees also block MmWave signals, so these signals cover a shorter distance. To resolve these issues, multiple small cell stations are installed to cover the gap between end-user and base station [18]. Small cell covers a very shorter range, so the installation of a small cell depends on the population of a particular area. Generally, in a populated place, the distance between each small cell varies from 10 to 90 meters. In the survey [20], various authors implemented small cells with massive MIMO simultaneously. They also reviewed multiple technologies used in 5G like beamforming, small cell, massive MIMO, NOMA, device to device (D2D) communication. Various problems like interference management, spectral efficiency, resource management, energy efficiency, and backhauling are discussed. The author also gave a detailed presentation of all the issues occurring while implementing small cells with various 5G technologies. As shown in the Figure 7, mmWave has a higher range, so it can be easily blocked by the obstacles as shown in Figure 7a. This is one of the key concerns of millimeter-wave signal transmission. To solve this issue, the small cell can be placed at a short distance to transmit the signals easily, as shown in Figure 7b.

### 5.2. Beamforming

Beamforming is a key technology of wireless networks which transmits the signals in a directional manner. 5G beamforming making a strong wireless connection toward a receiving end. In conventional systems when small cells are not using beamforming, moving signals to particular areas is quite difficult. Beamforming counter this issue using beamforming small cells are able to transmit the signals in particular direction towards a device like mobile phone, laptops, autonomous vehicle and IoT devices. Beamforming is improving the efficiency and saves the energy of the 5G network. Beamforming is broadly divided into three categories: Digital beamforming, analog beamforming and hybrid beamforming. Digital beamforming: multiuser MIMO is equal to digital beamforming which is mainly used in LTE Advanced Pro and in 5G NR. In digital beamforming the same frequency or time resources can be used to transmit the data to multiple users at the same time which improves the cell capacity of wireless networks. Analog Beamforming: In mmWave frequency range 5G NR analog beamforming is a very important approach which improves the coverage. In digital beamforming there are chances of high pathloss in mmWave as only one beam per set of antenna is formed. While the analog beamforming saves high pathloss in mmWave. Hybrid beamforming: hybrid beamforming is a combination of both analog beamforming and digital beamforming. In the implementation of MmWave in 5G network hybrid beamforming will be used [84].

**Figure 8:**
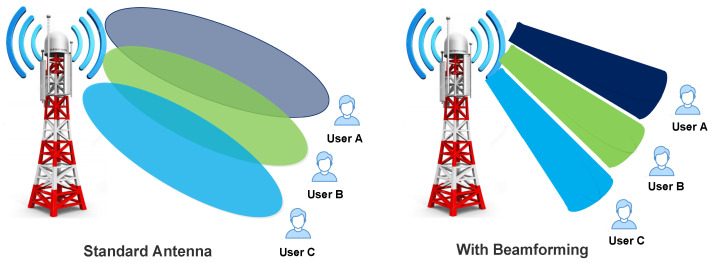
Pictorial Representation of communication with and without using beamforming.

Wireless signals in the 4G network are spreading in large areas, and nature is not Omnidirectional. Thus, energy depletes rapidly, and users who are accessing these signals also face interference problems. The beamforming technique is used in the 5G network to resolve this issue. In beamforming signals are directional. They move like a laser beam from the base station to the user, so signals seem to be traveling in an invisible cable. Beamforming helps achieve a faster data rate; as the signals are directional, it leads to less energy consumption and less interference. In [21], investigators evolve some techniques which reduce interference and increase system efficiency of the 5G mobile network. In this survey article, the authors covered various challenges faced while designing an optimized beamforming algorithm. Mainly focused on different design parameters such as performance evaluation and power consumption. In addition, they also described various issues related to beamforming like CSI, computation complexity, and antenna correlation. They also covered various research to cover how beamforming helps implement MIMO in next-generation mobile networks [85]. Figure 8 shows the pictorial representation of communication with and without using beamforming.

### 5.3. Mobile Edge Computing

**Figure 9:**
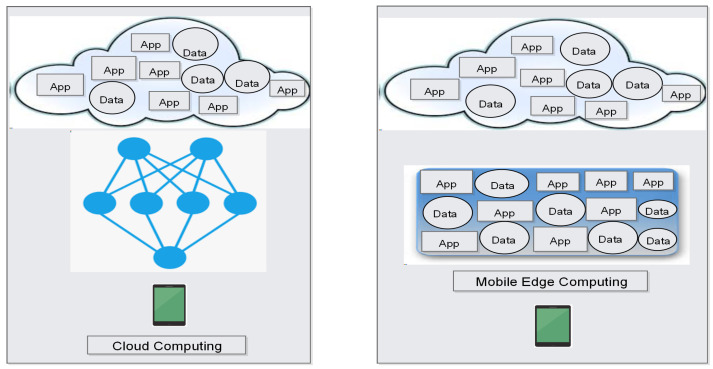
Pictorial representation of cloud computing vs. mobile edge computing.

Mobile Edge Computing (MEC) [24]: MEC is an extended version of cloud computing that brings cloud resources closer to the end-user. When we talk about computing, the very first thing that comes to our mind is cloud computing. Cloud computing is a very famous technology that offers many services to end-user. Still, cloud computing has many drawbacks. The services available in the cloud are too far from end-users that create latency, and cloud user needs to download the complete application before use, which also increases the burden to the device [86]. MEC creates an edge between the end-user and cloud server, bringing cloud computing closer to the end-user. Now, all the services, namely, video conferencing, virtual software, etc., are offered by this edge that improves cloud computing performance. Another essential feature of MEC is that the application is split into two parts, which, first one is available at cloud server, and the second is at the user’s device. Therefore, the user need not download the complete application on his device that increases the performance of the end user’s device. Furthermore, MEC provides cloud services at very low latency and less bandwidth. In [23,87], the author’s investigation proved that successful deployment of MEC in 5G network increases the overall performance of 5G architecture. Graphical differentiation between cloud computing and mobile edge computing is presented in Figure 9.

## 6. 5G Security

Security is the key feature in the telecommunication network industry, which is necessary at various layers, to handle 5G network security in applications such as IoT, Digital forensics, IDS and many more [88,89]. The authors [90], discussed the background of 5G and its security concerns, challenges and future directions. The author also introduced the blockchain technology that can be incorporated with the IoT to overcome the challenges in IoT. The paper aims to create a security framework which can be incorporated with the LTE advanced network, and effective in terms of cost, deployment and QoS. In [91], author surveyed various form of attacks, the security challenges, security solutions with respect to the affected technology such as SDN, Network function virtualization (NFV), Mobile Clouds and MEC, and security standardizations of 5G, i.e., 3GPP, 5GPPP, Internet Engineering Task Force (IETF), Next Generation Mobile Networks (NGMN), European Telecommunications Standards Institute (ETSI). In [92], author elaborated various technological aspects, security issues and their existing solutions and also mentioned the new emerging technological paradigms for 5G security such as blockchain, quantum cryptography, AI, SDN, CPS, MEC, D2D. The author aims to create new security frameworks for 5G for further use of this technology in development of smart cities, transportation and healthcare. In [93], author analyzed the threats and dark threat, security aspects concerned with SDN and NFV, also their Commercial & Industrial Security Corporation (CISCO) 5G vision and new security innovations with respect to the new evolving architectures of 5G [94].

AuthenticationThe identification of the user in any network is made with the help of authentication. The different mobile network generations from 1G to 5G have used multiple techniques for user authentication. 5G utilizes the 5G Authentication and Key Agreement (AKA) authentication method, which shares a cryptographic key between user equipment (UE) and its home network and establishes a mutual authentication process between the both [95].

Access Control To restrict the accessibility in the network, 5G supports access control mechanisms to provide a secure and safe environment to the users and is controlled by network providers. 5G uses simple public key infrastructure (PKI) certificates for authenticating access in the 5G network. PKI put forward a secure and dynamic environment for the 5G network. The simple PKI technique provides flexibility to the 5G network; it can scale up and scale down as per the user traffic in the network [96,97].

Communication Security 5G deals to provide high data bandwidth, low latency, and better signal coverage. Therefore secure communication is the key concern in the 5G network. UE, mobile operators, core network, and access networks are the main focal point for the attackers in 5G communication. Some of the common attacks in communication at various segments are Botnet, message insertion, micro-cell, distributed denial of service (DDoS), and transport layer security (TLS)/secure sockets layer (SSL) attacks [98,99].

Encryption The confidentiality of the user and the network is done using encryption techniques. As 5G offers multiple services, end-to-end (E2E) encryption is the most suitable technique applied over various segments in the 5G network. Encryption forbids unauthorized access to the network and maintains the data privacy of the user. To encrypt the radio traffic at Packet Data Convergence Protocol (PDCP) layer, three 128-bits keys are applied at the user plane, nonaccess stratum (NAS), and access stratum (AS) [100].

## 7. Summary of 5G Technology Based on Above-Stated Challenges

In this section, various issues addressed by investigators in 5G technologies are presented in Table 13. In addition, different parameters are considered, such as throughput, latency, energy efficiency, data rate, spectral efficiency, fairness & computing capacity, transmission rate, coverage, cost, security requirement, performance, QoS, power optimization, etc., indexed from R1 to R14.

## 8. Conclusions

This survey article illustrates the emergence of 5G, its evolution from 1G to 5G mobile network, applications, different research groups, their work, and the key features of 5G. It is not just a mobile broadband network, different from all the previous mobile network generations; it offers services like IoT, V2X, and Industry 4.0. This paper covers a detailed survey from multiple authors on different technologies in 5G, such as massive MIMO, Non-Orthogonal Multiple Access (NOMA), millimeter wave, small cell, MEC (Mobile Edge Computing), beamforming, optimization, and machine learning in 5G. After each section, a tabular comparison covers all the state-of-the-research held in these technologies. This survey also shows the importance of these newly added technologies and building a flexible, scalable, and reliable 5G network.

## 9. Future Findings

This article covers a detailed survey on the 5G mobile network and its features. These features make 5G more reliable, scalable, efficient at affordable rates. As discussed in the above sections, numerous technical challenges originate while implementing those features or providing services over a 5G mobile network. So, for future research directions, the research community can overcome these challenges while implementing these technologies (MIMO, NOMA, small cell, mmWave, beam-forming, MEC) over a 5G network. 5G communication will bring new improvements over the existing systems. Still, the current solutions cannot fulfill the autonomous system and future intelligence engineering requirements after a decade. There is no matter of discussion that 5G will provide better QoS and new features than 4G. But there is always room for improvement as the considerable growth of centralized data and autonomous industry 5G wireless networks will not be capable of fulfilling their demands in the future. So, we need to move on new wireless network technology that is named 6G. 6G wireless network will bring new heights in mobile generations, as it includes (i) massive human-to-machine communication, (ii) ubiquitous connectivity between the local device and cloud server, (iii) creation of data fusion technology for various mixed reality experiences and multiverps maps. (iv) Focus on sensing and actuation to control the network of the entire world. The 6G mobile network will offer new services with some other technologies; these services are 3D mapping, reality devices, smart homes, smart wearable, autonomous vehicles, artificial intelligence, and sense. It is expected that 6G will provide ultra-long-range communication with a very low latency of 1 ms. The per-user bit rate in a 6G wireless network will be approximately 1 Tbps, and it will also provide wireless communication, which is 1000 times faster than 5G networks.

## Figures and Tables

**Table 1:** Summary of Mobile Technology.

Generations	Access Techniques	Transmission Techniques	Error Correction Mechanism	Data Rate	Frequency Band	Bandwidth	Application	Description
1G	FDMA, AMPS	Circuit Switching	NA	2.4 kbps	800 MHz	Analog	Voice	Let us talk to each other
2G	GSM, TDMA, CDMA	Circuit Switching	NA	10 kbps	800 MHz, 900 MHz, 1800 MHz, 1900 MHz	25 MHz	Voice and Data	Let us send messages and travel with improved data services
3G	WCDMA, UMTS, CDMA 2000, HSUPA/HSDPA	Circuit and Packet Switching	Turbo Codes	384 kbps to 5 Mbps	800 MHz, 850 MHz, 900 MHz, 1800 MHz, 1900 MHz, 2100 MHz	25 MHz	Voice, Data, and Video Calling	Let us experience surfing internet and unleashing mobile applications
4G	LTEA, OFDMA, SCFDMA, WIMAX	Packet switching	Turbo Codes	100 Mbps to 200 Mbps	2.3 GHz, 2.5 GHz and 3.5 GHz initially	100 MHz	Voice, Data, Video Calling, HD Television, and Online Gaming.	Let’s share voice and data over fast broadband internet based on unified networks architectures and IP protocols
5G	BDMA, NOMA, FBMC	Packet Switching	LDPC	10 Gbps to 50 Gbps	1.8 GHz, 2.6 GHz and 30–300 GHz	30–300 GHz	Voice, Data, Video Calling, Ultra HD video, Virtual Reality applications	Expanded the broadband wireless services beyond mobile internet with IOT and V2X.

**Table 2:** Table of Notations and Abbreviations.

Abbreviation	Full Form	Abbreviation	Full Form
AMF	Access and Mobility Management Function	M2M	Machine-to-Machine
AT&T	American Telephone and Telegraph	mmWave	millimeter wave
BS	Base Station	NGMN	Next Generation Mobile Networks
CDMA	Code-Division Multiple Access	NOMA	Non-Orthogonal Multiple Access
CSI	Channel State Information	NFV	Network Functions Virtualization
D2D	Device to Device	OFDM	Orthogonal Frequency Division Multiplexing
EE	Energy Efficiency	OMA	Orthogonal Multiple Access
EMBB	Enhanced mobile broadband:	QoS	Quality of Service
ETSI	European Telecommunications Standards Institute	RNN	Recurrent Neural Network
eMTC	Massive Machine Type Communication	SDN	Software-Defined Networking
FDMA	Frequency Division Multiple Access	SC	Superposition Coding
FDD	Frequency Division Duplex	SIC	Successive Interference Cancellation
GSM	Global System for Mobile	TDMA	Time Division Multiple Access
HSPA	High Speed Packet Access	TDD	Time Division Duplex
IoT	Internet of Things	UE	User Equipment
IETF	Internet Engineering Task Force	URLLC	Ultra Reliable Low Latency Communication
LTE	Long-Term Evolution	UMTC	Universal Mobile Telecommunications System
ML	Machine Learning	V2V	Vehicle to Vehicle
MIMO	Multiple Input Multiple Output	V2X	Vehicle to Everything

**Table 3:** A comparative overview of existing surveys on different technologies of 5G networks.

Authors& References	MIMO	NOMA	MmWave	5G IOT	5G ML	Small Cell	Beamforming	MEC	5G Optimization
Chataut and Akl [7]	Yes	-	Yes	-	-	-	Yes	-	-
Prasad et al. [8]	Yes	-	Yes	-	-	-	-	-	-
Kiani and Nsari [9]	-	Yes	-	-	-	-	-	Yes	-
Timotheou and Krikidis [10]	-	Yes	-	-	-	-	-	-	Yes
Yong Niu et al. [11]	-	-	Yes	-	-	Yes	-	-	-
Qiao et al. [12]	-	-	Yes	-	-	-	-	-	Yes
Ramesh et al. [13]	Yes	-	Yes	-	-	-	-	-	-
Khurpade et al. [14]	Yes	Yes	-	Yes	-	-	-	-	-
Bega et al. [15]	-	-	-	-	Yes	-	-	-	Yes
Abrol and jha [16]	-	-	-	-	-	Yes	-	-	Yes
Wei et al. [17]	-	Yes		-	-	-	-	-	-
Jakob Hoydis et al. [18]	-	-	-	-	-	Yes	-	-	-
Papadopoulos et al. [19]	Yes	-	-	-	-	-	Yes	-	-
Shweta Rajoria et al. [20]	Yes	-	Yes	-	-	Yes	Yes	-	-
Demosthenes Vouyioukas [21]	Yes	-	-	-	-	-	Yes	-	-
Al-Imari et al. [22]	-	Yes	Yes	-	-	-	-	-	-
Michael Till Beck et al. [23]	-	-	-	-	-	-		Yes	-
Shuo Wang et al. [24]	-	-	-	-	-	-		Yes	-
Gupta and Jha [25]	Yes	-	-	-	-	Yes	-	Yes	-
Our Survey	Yes	Yes	Yes	Yes	Yes	Yes	Yes	Yes	Yes

**Table 4:** Research groups working on 5G mobile networks.

Research Groups	Research Area	Description
METIS (Mobile and wireless communications Enablers for Twenty-twenty (2020) Information Society)	Working 5G Framework	METIS focused on RAN architecture and designed an air interface which evaluates data rates on peak hours, traffic load per region, traffic volume per user and actual client data rates. They have generate METIS published an article on February, 2015 in which they developed RAN architecture with simulation results. They design an air interface which evaluates data rates on peak hours, traffic load per region, traffic volume per user and actual client data rates.They have generate very less RAN latency under 1ms. They also introduced diverse RAN model and traffic flow in different situation like malls, offices, colleges and stadiums.
5G PPP (5G Infrastructure Public Private Partnership)	Next generation mobile network communication, high speed Connectivity.	Fifth generation infrastructure public partnership project is a joint startup by two groups (European Commission and European ICT industry). 5G-PPP will provide various standards architectures, solutions and technologies for next generation mobile network in coming decade. The main motto behind 5G-PPP is that, through this project, European Commission wants to give their contribution in smart cities, e-health, intelligent transport, education, entertainment, and media.
5GNOW (5th Generation Non-Orthogonal Waveforms for asynchronous signaling)	Non-orthogonal Multiple Access	5GNOW’s is working on modulation and multiplexing techniques for next generation network. 5GNOW’s offers ultra-high reliability and ultra-low latency communication with visible waveform for 5G. 5GNOW’s also worked on acquiring time and frequency plane information of a signal using short term Fourier transform (STFT)
EMPhAtiC (Enhanced Multicarrier Technology for Professional Ad-Hoc and Cell-Based Communications)	MIMO Transmission	EMPhAtiC is working on MIMO transmission to develop a secure communication techniques with asynchronicity based on flexible filter bank and multihop. Recently they also launched MIMO based trans-receiver technique under frequency selective channels for Filter Bank Multi-Carrier (FBMC)
NEWCOM (Network of Excellence in Wireless Communications)	Advanced aspects of wireless communications	NEWCOM is working on energy efficiency, channel efficiency, multihop communication in wireless communication. Recently, they are working on cloud RAN, mobile broadband, local and distributed antenna techniques and multi-hop communication for 5G network. Finally, in their final research they give on result that QAM modulation schema, system bandwidth and resource block is used to process the base band.
NYU New York University Wireless	Millimeter Wave	NYU Wireless is research center working on wireless communication, sensors, networking and devices. In their recent research, NYU focuses on developing smaller and lighter antennas with directional beamforming to provide reliable wireless communication.
5GIC 5G Innovation Centre	Decreasing network costs, Preallocation of resources according to user’s need, point-to-point communication, Highspeed connectivity.	5GIC, is a UK’s research group, which is working on high-speed wireless communication. In their recent research they got 1Tbps speed in point-to-point wireless communication. Their main focus is on developing ultra-low latency app services.
ETRI (Electronics and Telecommunication Research Institute)	Device-to-device communication, MHN protocol stack	ETRI (Electronics and Telecommunication Research Institute), is a research group of Korea, which is focusing on improving the reliability of 5G network, device-to-device communication and MHN protocol stack.

**Table 5:** Summary of massive MIMO-based approaches in 5G technology.

Approach	Throughput	Latency	Energy Efficiency	Spectral Efficiency
Panzner et al. [47]	Good	Low	Good	Average
He et al. [48]	Average	Low	Average	-
Prasad et al. [8]	Good	-	Good	Avearge
Papadopoulos et al. [19]	Good	Low	Average	Avearge
Ramesh et al. [13]	Good	Average	Good	Good
Zhou et al. [51]	Average	-	Good	Average

**Table 6:** Summary of NOMA-based approaches in 5G technology.

Approach	Spectral Efficiency	Fairness	Computing Capacity
Al-Imari et al. [22]	Good	Good	Average
Islam et al. [53]	Good	Average	Average
Kiani and Nsari [9]	Average	Good	Good
Timotheou and Krikidis [10]	Good	Good	Average
Wei et al. [17]	Good	Average	Good

**Table 7:** Summary of existing mmWave-based approaches in 5G technology.

Approach	Transmission Rate	Coverage	Cost
Hong et al. [61]	Average	Average	Low
Qiao et al. [12]	Average	Good	Average
Wei et al. [62]	Good	Average	Low

**Table 8:** Summary of IoT-based approaches in 5G technology.

Approach	Data Rate	Security Requirement	Performance
Akpakwu et al. [65]	Good	Average	Good
Khurpade et al. [14]	Average	-	Average
Ni et al. [66]	Good	Average	Average

**Table 9:** The state-of-the-art ML-based solution for 5G network.

Author References	Key Contribution	ML Applied	Network Participants Component		5G Network Application Parameter				
			**RAN**	**Core**	**LB**	**SDN**	**RAN**	**RA**	**SEC**
Alave et al. [68]	Network traffic prediction	LSTM and DNN	✓	✓	*	✓	✓	✓	X
Bega et al. [15]	Network slice admission control algorithm	Machine Learning and Deep Learing	✓	X	X	✓	✓	✓	X
Suomalainen et al. [69]	5G Security	Machine Learning	X	✓	✓	✓	✓	✓	✓
Bashir et al. [70]	Resource Allocation	Machine Learning	✓	✓	✓	✓	✓	✓	X
Balevi et al. [71]	Low Latency communication	Unsupervised clustering	X	✓	X	✓	✓	✓	X
Tayyaba et al. [72]	Resource Management	LSTM, CNN, and DNN	✓	✓	X	✓	✓	✓	✓
Sim et al. [73]	5G mmWave Vehicular communication	FML (Fast machine Learning)	X	✓	*	✓	✓	✓	X
Li et al. [74]	Intrusion Detection System	Machine Learning	X	✓	X	✓	✓	✓	✓
Kafle et al. [75]	5G Network Slicing	Machine Learning	X	✓	X	✓	✓	✓	✓
Chen et al. [76]	Physical-Layer Channel Authentication	Machine Learning	X	✓	X	X	X	X	✓
Sevgican et al. [77]	Intelligent Network Data Analytics Function in 5G	Machine Learning	✓	X	✓	X	X	*	*
Abidi et al. [78]	Optimal 5G network slicing	Machine Learning and Deep Learing	X	✓	X	✓	✓	✓	*

**Table 10:** The state-of-the-art ML-based approaches in 5G technology.

Approach	Energy Efficiency	Quality of Services (QoS)	Latency
Fang et al. [79]	Good	Good	Average
Alawe et al. [68]	Good	Average	Low
Bega et al. [15]	-	Good	Average

**Table 11:** Summary of Optimization Based Approaches in 5G Technology.

Approach	Energy Efficiency	Power Optimization	Latency
Zi et al. [80]	Good	-	Average
Abrol and jha [16]	Good	Good	-
Pérez-Romero et al. [81]	-	Average	Average
Lähetkangas et al. [82]	Average	-	Low

**Table 12:** Types of Small cells.

Types of Small Cell	Coverage Radius	Indoor Outdoor	Transmit Power	Number of Users	Backhaul Type	Cost
Femtocells	30–165 ft 10–50 m	Indoor	100 mW 20 dBm	8–16	Wired, fiber	Low
Picocells	330–820 ft 100–250 m	Indoor Outdoor	250 mW 24 dBm	32–64	Wired, fiber	Low
Microcells	1600–8000 ft 500–250 m	Outdoor	2000–500 mW 32–37 dBm	200	Wired, fiber, Microwave	Medium

**Table 13:** Summary of 5G Technology above stated challenges (R1:Throughput, R2:Latency, R3:Energy Efficiency, R4:Data Rate, R5:Spectral efficiency, R6:Fairness & Computing Capacity, R7:Transmission Rate, R8:Coverage, R9:Cost, R10:Security requirement, R11:Performance, R12:Quality of Services (QoS), R13:Power Optimization).

Approach	R1	R2	R3	R4	R5	R6	R7	R8	R9	R10	R11	R12	R13	R14
Panzner et al. [47]	Good	Low	Good	-	Avg	-	-	-	-	-	-	-	-	-
Qiao et al. [12]	-	-	-	-	-	-	-	Avg	Good	Avg	-	-	-	-
He et al. [48]	Avg	Low	Avg	-	-	-	-	-	-	-	-	-	-	-
Abrol and jha [16]	-	-	Good	-	-	-	-	-	-	-	-	-	-	Good
Al-Imari et al. [22]	-	-	-	-	Good	Good	Avg	-	-	-	-	-	-	-
Papadopoulos et al. [19]	Good	Low	Avg	-	Avg	-	-	-	-	-	-	-	-	-
Kiani and Nsari [9]	-	-	-	-	Avg	Good	Good	-	-	-	-	-	-	-
Beck [23]	-	Low	-	-	-	-	-	Avg	-	-	-	Good	-	Avg
Ni et al. [66]	-	-	-	Good	-	-	-	-	-	-	Avg	Avg	-	-
Elijah [50]	Avg	Low	Avg	-	-	-	-	-	-	-	-	-	-	-
Alawe et al. [68]	-	Low	Good	-	-	-	-	-	-	-	-	-	Avg	-
Zhou et al. [51]	Avg	-	Good	-	Avg	-	-	-	-	-	-	-	-	-
Islam et al. [53]	-	-	-	-	Good	Avg	Avg	-	-	-	-	-	-	-
Bega et al. [15]	-	Avg	-	-	-	-	-	-	-	-	-	-	Good	-
Akpakwu et al. [65]	-	-	-	Good	-	-	-	-	-	-	Avg	Good	-	-
Wei et al. [17]	-	-	-	-	-	-	-	Good	Avg	Low	-	-	-	-
Khurpade et al. [14]	-	-	-	Avg	-	-	-	-	-	-	-	Avg	-	-
Timotheou and Krikidis [10]	-	-	-	-	Good	Good	Avg	-	-	-	-	-	-	-
Wang [45]	Avg	Low	Avg	Avg	-	-	-	-	-	-	-	-	-	-
Akhil Gupta & R. K. Jha [25]	-	-	Good	Avg	Good	-	-	-	-	-	-	Good	Good	-
Pérez-Romero et al. [81]	-	-	Avg	-	-	-	-	-	-	-	-	-	-	Avg
Pi [59]	-	-	-	-	-	-	-	Good	Good	Avg	-	-	-	-
Zi et al. [80]	-	Avg	Good	-	-	-	-	-	-	-	-	-	-	-
Chin [101]	-	-	Good	Avg	-	-	-	-	-	Avg	-	Good	-	-
Mamta Agiwal [5]	-	Avg	-	Good	-	-	-	-	-	-	Good	Avg	-	-
Ramesh et al. [13]	Good	Avg	Good	-	Good	-	-	-	-	-	-	-	-	-
Niu [11]	-	-	-	-	-	-	-	Good	Avg	Avg	-	-	-	
Fang et al. [79]	-	Avg	Good	-	-	-	-	-	-	-	-	-	Good	-
Hoydis [18]	-	-	Good	-	Good	-	-	-	-	Avg	-	Good	-	-
Wei et al. [62]	-	-	-	-	Good	Avg	Good	-	-	-	-	-	-	-
Hong et al. [61]	-	-	-	-	-	-	-	-	Avg	Avg	Low	-	-	-
Rashid [102]	-	-	-	Good	-	-	-	Good	-	-	-	Avg	-	Good
Prasad et al. [8]	Good	-	Good	-	Avg	-	-	-	-	-	-	-	-	-
Lähetkangas et al. [82]	-	Low	Av	-	-	-	-	-	-	-	-	-	-	-

## Data Availability

Not applicable.
